# Artificial intelligence in radiology: does it impact medical students preference for radiology as their future career?

**DOI:** 10.1259/bjro.20200037

**Published:** 2020-12-11

**Authors:** Abdulmajeed Bin Dahmash, Mohammed Alabdulkareem, Aljabriyah Alfutais, Ahmed M Kamel, Feras Alkholaiwi, Shaker Alshehri, Yousof Al Zahrani, Mohammed Almoaiqel

**Affiliations:** 1 College of Medicine, Imam Mohammad ibn Saud Islamic University, Riyadh, Saudi Arabia; 2 Neuroradiology Division, Department of Medical Imaging, King Abdulaziz Medical City & King Abdullah Specialized Children’s Hospital, Ministry of National Guard-Health Affairs, Riyadh, Saudi Arabia; 3 Vascular and Interventional Radiology Unit, Department of Medical Imaging, King Abdulaziz Medical City & King Abdullah Specialized Children’s Hospital, Ministry of National Guard-Health Affairs, Riyadh, Saudi Arabia; 4 Department of Clinical Pharmacy, Faculty of Pharmacy, Cairo University, Cairo, Egypt

## Abstract

**Objective:**

To test medical students’ perceptions of the impact of artificial intelligence (AI) on radiology and the influence of these perceptions on their choice of radiology as a lifetime career.

**Methods:**

A cross-sectional multicenter survey of medical students in Saudi Arabia was conducted in April 2019.

**Results:**

Of the 476 respondents, 34 considered radiology their first specialty choice, 26 considered it their second choice, and 65 considered it their third choice. Only 31% believed that AI would replace radiologists in their lifetime, while 44.8% believed that AI would minimize the number of radiologists needed in the future. Approximately 50% believed they had a good understanding of AI; however, when knowledge of AI was tested using five questions, on average, only 22% of the questions were answered correctly. Among the respondents who ranked radiology as their first choice, 58.8% were anxious about the uncertain impact of AI on radiology. The number of respondents who ranked radiology as one of their top three choices increased by 14 when AI was not a consideration. Radiology conferences and the opinions of radiologists had the most influence on the respondents’ preferences for radiology.

**Conclusion:**

The worry that AI might displace radiologists in the future had a negative influence on medical students’ consideration of radiology as a career. Academic radiologists are encouraged to educate their students about AI and its potential impact when students are considering radiology as a lifetime career choice.

**Advances in knowledge:**

Rapid advances of AI in radiology will certainly impact the specialty, the concern of AI impact on radiology had negative influence in our participants and investing in AI education and is highly recommended.

## Introduction

Over the past decade, artificial intelligence (AI) and machine learning (ML) have become some of the most highly discussed topics in radiology, with about 800 related publications in 2017 alone.^1^ AI is a scientific field of computer skills that can function similar to a human being intelligence in learning ability and problem-solving skills, the more images radiology residents see, the more he/she learn, and the same applies to ML and deep learning (DL), ML is a branch of AI in which an algorithm allows computers to continuously advance based on data without manual programming, and it is the most commonly used type of AI in radiology.^1–3^ Rapid advances in the field of radiology will certainly change the practice of radiologists,^1^ as routine tasks can be performed faster and more efficiently with the aid of AI.^4,5^ However, parts of radiologists’ jobs can be complicated for AI, such as resolving complicated clinical cases.^4,5^ One paper from the European Society of Radiology indicated that AI will not replace radiologists; in fact, it will improve radiology and increase radiologists’ value and importance.^4,6^ However, radiologists need to educate themselves on AI and work with AI researchers to ensure that AI is used in the best way possible to provide benefit to patients.^4,6^ Similarly, other healthcare specialties will be also impacted by AI such as pathology, dermatology, ophthalmology, cardiology and others.^7^

Radiology is an appealing specialty to many medical students for various reasons, including the lifestyle of diagnostic radiology and diverse sub-specialty options for diagnostic and interventional radiology. In Saudi Arabia, radiologists make $210,000 on average annually in private practice with an average of 0.04 radiologists per 1000 citizens, Canada has the highest estimated average income for radiologists with $700,000 annually and an average of 0.07 radiologists per 1000 citizens.^8^ In addition, radiologists in the USA make $427,000 annually on average, about $170,000 more than other specialties, like pediatrics, internal medicine, and family medicine.^9,10^ Interest in radiology in the USA increased from 1990 on, reaching its peak in 2009, when 7% of senior medical students in the USA applied for radiology residency positions.^9,11^ Since then, the interest in radiology has been declining.^9,11^ To the authors knowledge, there are no published statistics in Saudi Arabia for the number of applications to radiology to measure if the interest in radiology has increased or declined for the past years.

In Abduljabbar et al study in Saudi Arabia on factors influencing medical students on their choice for radiology as a career, it is demonstrated that high salary, fewer working hours and job flexibility were the main reasons to choose radiology among their respondents, however, no direct patient contact and use of growing technology were the main reasons for not choosing radiology.^12^ In 2016, Geoffrey Hinton, a famous computer scientist, said “we should stop training radiologists now; it is just completely obvious deep learning is going to do better than radiologists”.^13^ This statement attracted attention in the general media, among radiologists, and in scientific radiology journals.^9,14–18^ Misunderstanding about the effect of AI on radiology may discourage medical students from considering radiology as a specialty.^14^ Previous studies in the same subject from Canada,^14^ Germany,^19^ UK^20^, and Brazil^21^ concluded that AI played a negative role in medical students’ choice for radiology.

Medical students need to understand AI and its implications in the field of radiology in order to make a rational decision about radiology as a future career. The aim of this study is to assess medical students’ perception of AI in radiology and the impact of these perceptions on their choice regarding radiology as a career.

## Methods

In April 2019, a quantitative, cross-sectional, survey-based study was conducted among medical students in three different medical schools in Riyadh, Saudi Arabia. Students from each university were approached by their group leaders, who volunteered for this study and sent e-mails to the students containing a link to the survey, an explanation of the purpose of the survey, and a request for consent to participate. The survey was sent to all students in their clinical years at three different universities. Students in their pre-clinical years were excluded from this study since they have not had enough exposure to all medical specialties. The anticipated population in these three schools on their clinical year at the time of this study is 1200, 476 responses received for a response rate of 39.6%. Institutional Review Board approval was obtained in advance from medical research center, college of medicine, Imam Mohammed ibn Saud Islamic University, Riyadh, Saudi Arabia.

A questionnaire previously developed by Gong et al^14^ was used to test students’ perceptions of the impact of AI and its influence on their choice regarding radiology as a career. The first part of the survey included questions about the students’ sex, interest in radiology, interest in radiology without considering AI, and exposure to radiology. The second part of the survey assessed students’ perceptions of the impact of AI on radiology, which were measured using a 7-point Likert scale ranging from 1 (strongly disagree) to 7 (strongly agree). It also included a question assessing the students’ exposure to AI. The third part of the survey assessed the students’ understanding of deep learning (DL) using five true/false questions. The last part of the survey included questions about the sources from which students obtained information on AI and their opinions about what the radiology community can do to advise medical students regarding the impact of AI.

Statistical analysis was performed using RStudio v.1.2 (RStudio, Inc.: Boston, MA, USA). Descriptive statistics were obtained using counts and percentages for categorical and ordinal items. Some ordinal items (*e.g.,* knowledge regarding AI and perceived understanding of AI) were also summarized using medians and interquartile ranges (IQRs). For sources of information about AI and radiology exposure, percentages were calculated for each source based on the total number of valid responses. Analysis was performed using the chi-square test of independence, Mann–Whitney rank sum test, and Kruskal–Wallis test. The chi-square test of independence was used to assess whether the distribution of responses regarding future specialty choice significantly differed based on whether the potential impact of AI was considered or not. Perceptions of the potential impact of AI on the field of radiology were assessed using four items measured by a 7-point Likert scale. Demographical characteristic analysis was performed for the whole study sample. Further analysis was restricted to participants who ranked radiology as one of their top three specialty choices. For the chi-square test performed to assess the association between anxiety regarding AI and demographic characteristics, the Likert responses “strongly agree,” “agree,” and “somewhat agree” were combined into one group, while the remaining four options were combined into a second group. The Mann–Whitney rank sum and Kruskal–Wallis tests were used to assess whether the mean rank of ordinal data significantly differed between the groups. The Mann–Whitney test was used in cases of two groups, while the Kruskal–Wallis test was used in cases of more than two groups. Hypothesis testing was performed at a 5% level of significance.

## Results

The study sample included 476 respondents (60.5% males and 39.5% females). The consideration of radiology as a specialty varied between respondents; 7.14% considered radiology as their first choice, 5.46% considered it as a second choice, and 13.7% considered it as a third choice.

The relevant response rate was defined as the percentage of respondents who ranked radiology as one of their top three choices. In the current analysis, 125 respondents ranked radiology as such. Thus, the relevant response rate was 125*100/476 = 26.26%.

**Figure 1.**
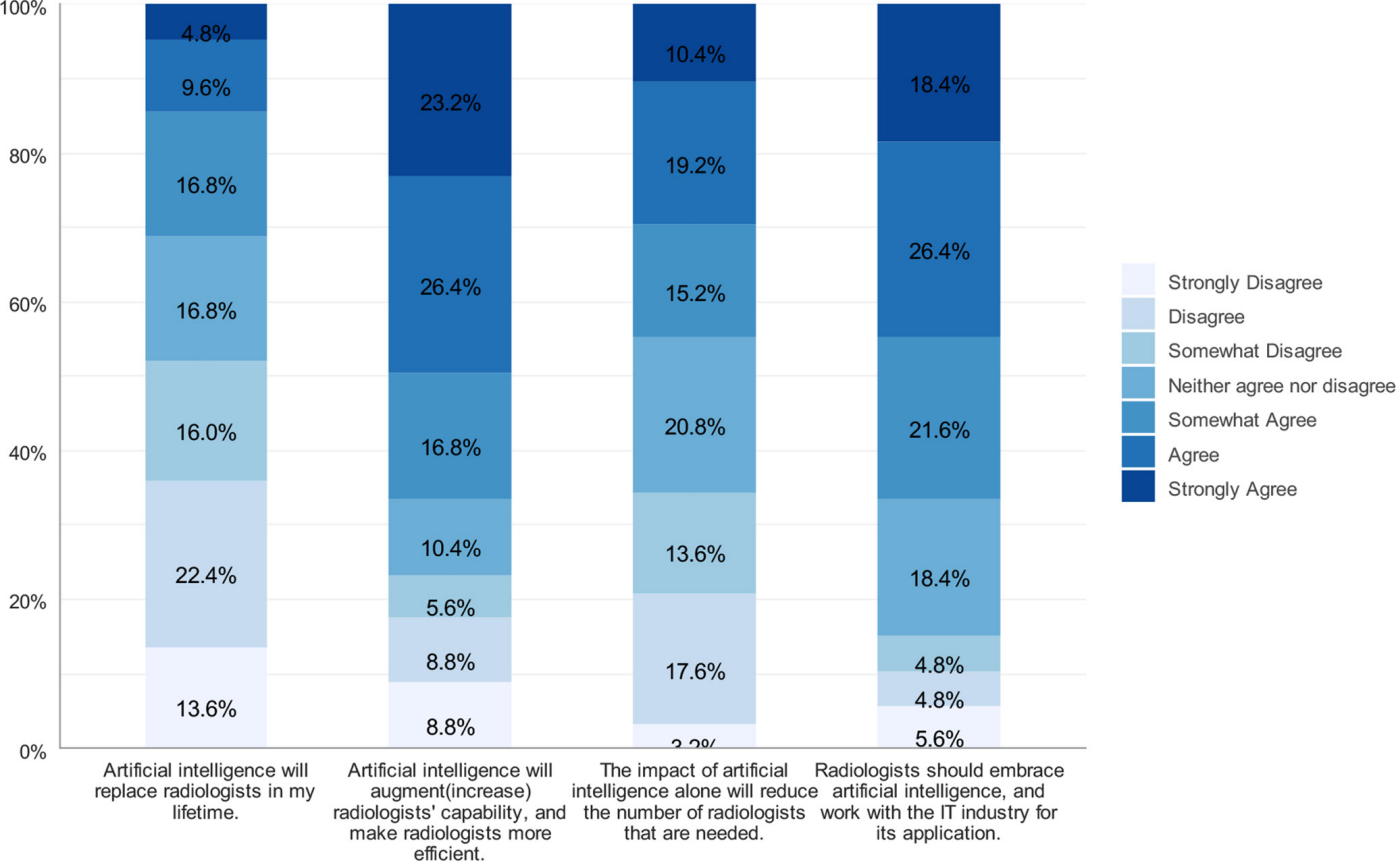
Interested students’ perceptions of the potential impact of AI

Respondents’ perceptions of the potential impact of AI on radiology are shown in (Figure 1). The analysis was restricted to the 125 respondents who chose radiology as their first, second, or third career option. When asked whether AI will replace radiologists during the participants’ lifetime, 31.2% agreed (defined as the combined percentage of “strongly agree,” “agree,” or “somewhat agree”), while 52% disagreed (defined as the combined percentage of “strongly disagree,” “disagree,” or “somewhat disagree”). Nearly 50% of the respondents agreed that AI will reduce the number of radiologists that are needed, while 34.4% disagreed.

**Figure 2.**
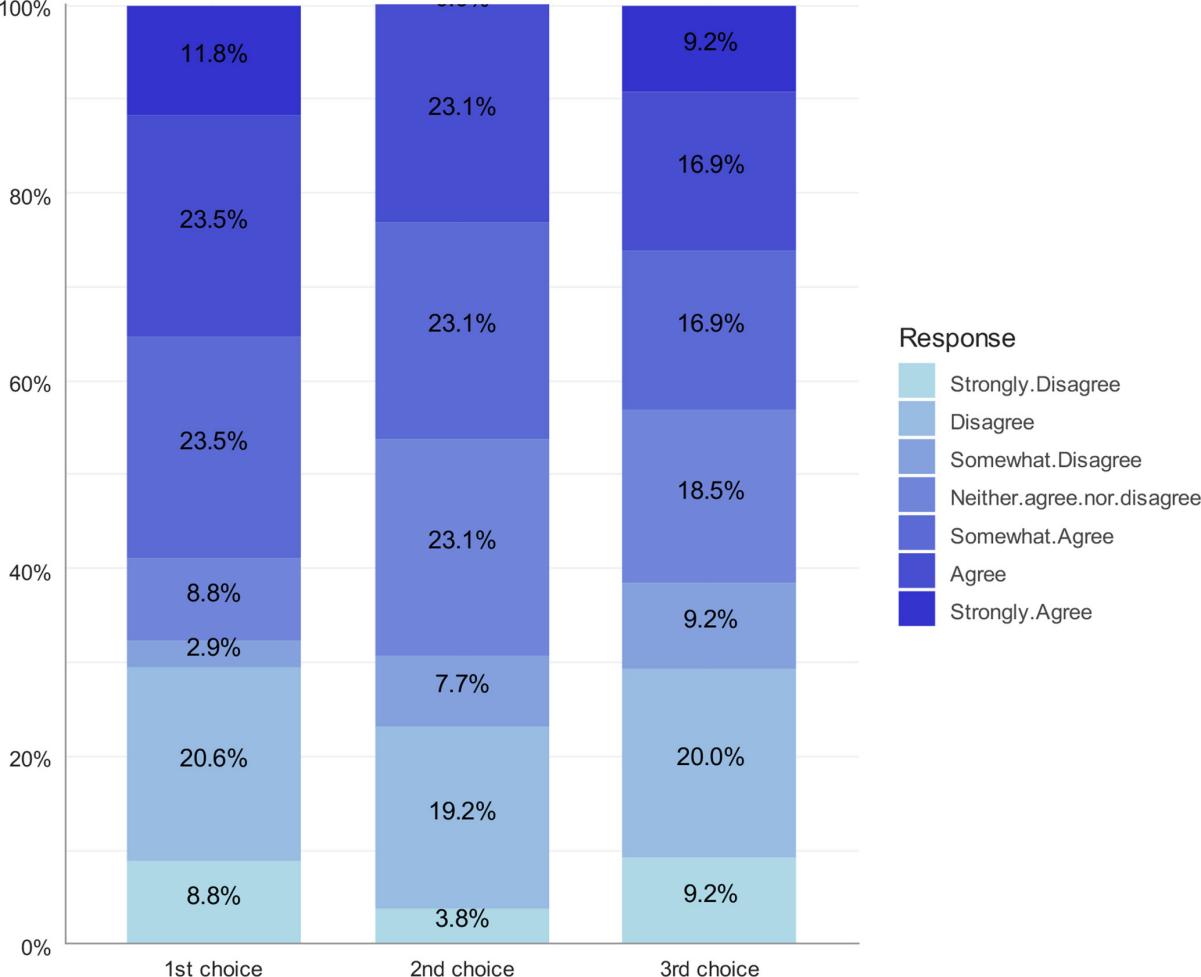
Respondents ranking radiology as their top three choices answer to “The uncertain impact of AI makes me worried to choose radiology as my career.”

The Kruskal–Wallis test did not show a statistically significant association between anxiety regarding the use of AI in radiology and respondents’ ranking of radiology as one of their top three choices (*p* = 0.9378). Anxiety regarding the use of AI in radiology among respondents who ranked radiology as one of their top three choices is shown in Figure 2.

Participants’ understanding of AI was assessed using based on self-reported confidence (one question) and knowledge about AI (five questions). The whole sample (*n* = 476) was used to ensure that the assessment was not affected by the participants’ specialty preference. The results showed that ~50% of the participants agreed (defined as choosing “strongly agree,” “agree,” or “somewhat agree”) that they have a good understanding of AI. The remaining 50% chose “neutral” (17.6%), “somewhat disagree” (10.5%), “disagree” (14.3%), or “strongly disagree” (6.3%).

Knowledge of AI was assessed using five questions for which participants could answer “true,” “false,” or “I don’t know.” The percentage of correct answers did not exceed 25% for four of the questions (Table 1). In addition, 259 (54.4%) of the included medical students did not answer any of the five questions correctly. This indicates that AI knowledge is low among medical students.

**Table 1.** Knowledge towards AI among all respondents using an objective assessment (*n* = 476)

AI-deep learning objective assessment questions presents as true/false and I don’t know	Correct answers
Deep learning is a class of machine learning algorithms that use multiple layers of neural networks.	19.5%
Deep learning methods learn directly from data, without the need of hand-engineered feature extraction.	18.3%
Application of deep learning in radiology requires large databases of labeled medical images.	33.6%
Deep learning systems are often opaque: it can be difficult to delineate the underlying “thought process”.	17.9%
Existing deep learning technology can achieve good pattern recognition but lacks the ability of deductive reasoning.	22.1%

Statistical analysis was performed using the chi-square test to assess the factors associated with greater understanding of AI. Participants were divided into two groups: those who did not answer any question correctly (Group 1) and those who answered at least one question correctly (Group 2). Participants who were interested in diagnostic radiology were more prevalent in Group two than in Group 1 (19.8% *vs* 11.6%). Exposure to AI was associated with answering at least one question correctly (*p* < 0.001). Participants who were exposed to AI were more prevalent in Group 2 (low anxiety) compared to Group 1 (41.9% *vs* 18.5%; see Table 2).

**Table 2.** Factors associated with knowledge towards AI

	Group 1 *N* = 259	Group 2 *N* = 217	*P*
**Interest**			**0.014**
More interested in Diagnostic Radiology	30 (11.6%)	43 (19.8%)	
Equally interested	33 (12.7%)	33 (15.2%)	
More interested in Interventional Radiology	91 (35.1%)	81 (37.3%)	
Not interested in Radiology	71 (27.4%)	45 (20.7%)	
Unsure	34 (13.1%)	15 (6.91%)	
**Exposure to AI:**			**<0.001**
No	211 (81.5%)	126 (58.1%)	
Yes	48 (18.5%)	91 (41.9%)	
**Exposure to radiology:**			0.673
No	28 (10.8%)	20 (9.22%)	
Yes	231 (89.2%)	197 (90.8%)	

Statistical analysis was performed using Chi-square test of independence

We analyzed responses from the 139 respondents who ranked radiology as one of their top three specialty choices if the potential impact of AI was not taken into consideration. Students were divided into two groups. Group one includes those who agreed (“somewhat agree,” “agree,” or “strongly agree”) that they had anxiety towards AI, and Group two includes those who did not agree (“neither agree nor disagree,” “somewhat disagree,” “disagree,” or “strongly disagree”). Only respondents’ self-perception of AI understanding was significantly associated with anxiety towards AI (*p* = 0.037 using Mann–Whitney test): participants who showed anxiety towards the use of AI had a significantly higher self-perceived understanding of AI compared to those who did not show anxiety (Table 3).

**Table 3.** Factors associated with anxiety towards the use of AI

	No anxiety *N* = 66	Anxiety *N* = 73	*P*
**Gender:**			0.398
Female	19 (28.8%)	27 (37.0%)	
Male	47 (71.2%)	46 (63.0%)	
**Interest:**			0.362
More interested in Diagnostic Radiology	18 (27.3%)	22 (30.1%)	
Equally interested	15 (22.7%)	18 (24.7%)	
More interested in Interventional Radiology	32 (48.5%)	27 (37.0%)	
Not interested in Radiology	0 (0.00%)	1 (1.37%)	
Unsure	1 (1.52%)	5 (6.85%)	
**Exposure to AI:**			0.364
No	43 (65.2%)	41 (56.2%)	
Yes	23 (34.8%)	32 (43.8%)	
**Exposure to radiology:**			0.668
No	3 (4.55%)	2 (2.74%)	
Yes	63 (95.5%)	71 (97.3%)	
**Self-perception of AI understanding**	4.00 [3.00;6.00]	5.00 [4.00;6.00]	**0.037**

Statistical analysis was performed using Mann-Whitney test for self-perception of AI understanding and Chi-square test of independence for all remaining variables

**Figure 3.**
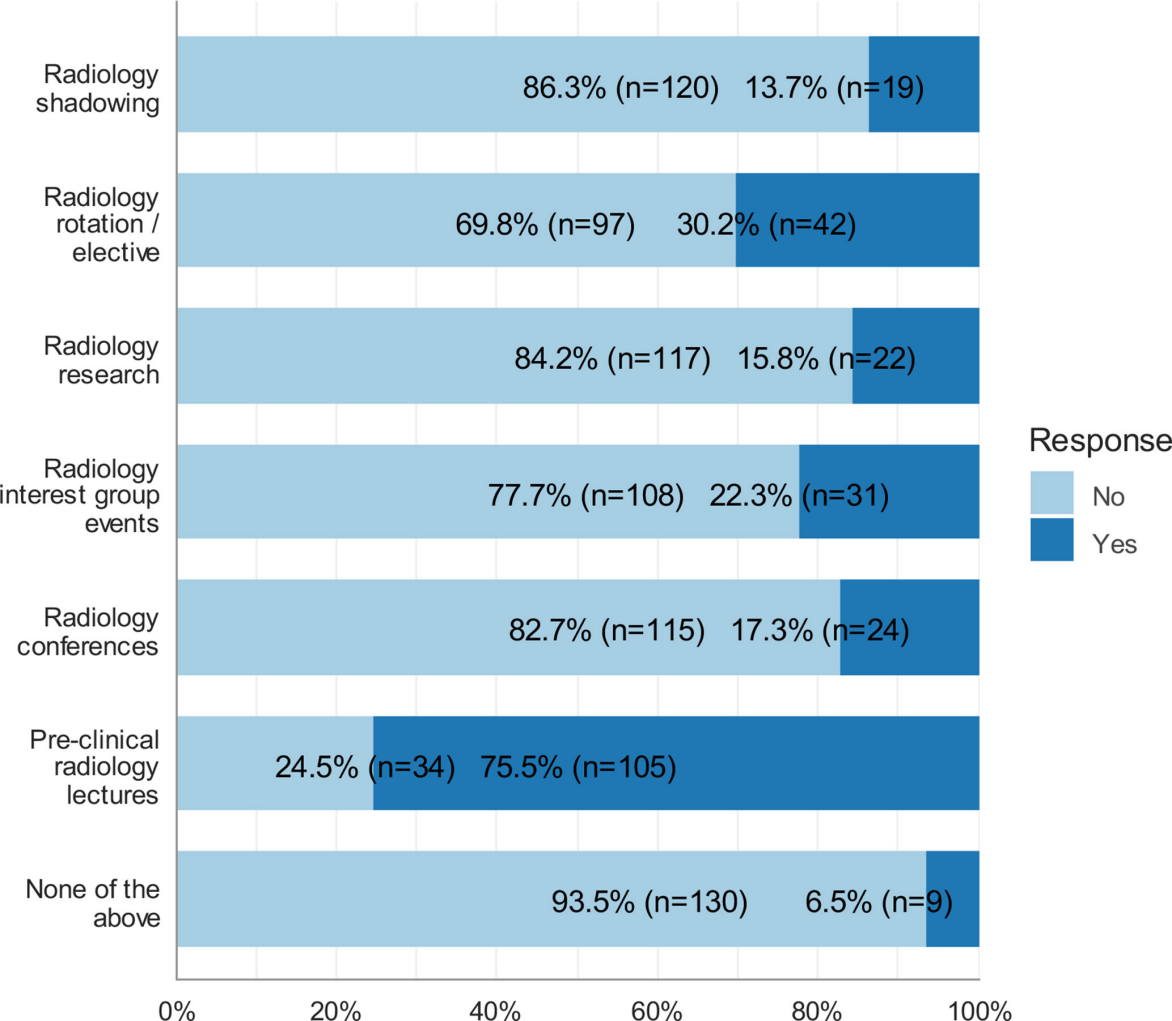
Sources of exposure to Radiology, only participants who ranked radiology as one of their top three choices were included in the analysis (*n* = 139).

Figure 3 shows the sources through which participants were exposed to radiology. Only participants who ranked radiology as one of their top three choices were included in the analysis (*n* = 139). Pre-clinical radiology lectures were the most prevalent source of exposure to radiology (*n* = 105, 75.5%), followed by radiology rotations/electives (*n* = 42, 30.2%).

The majority of respondents mentioned that they were not exposed to AI (*n* = 86, 61.9%). Exposure did not exceed 20% for any of the three sources (radiology research involving AI, courses on AI/machine learning, and computer science projects involving AI).

**Figure 4.**
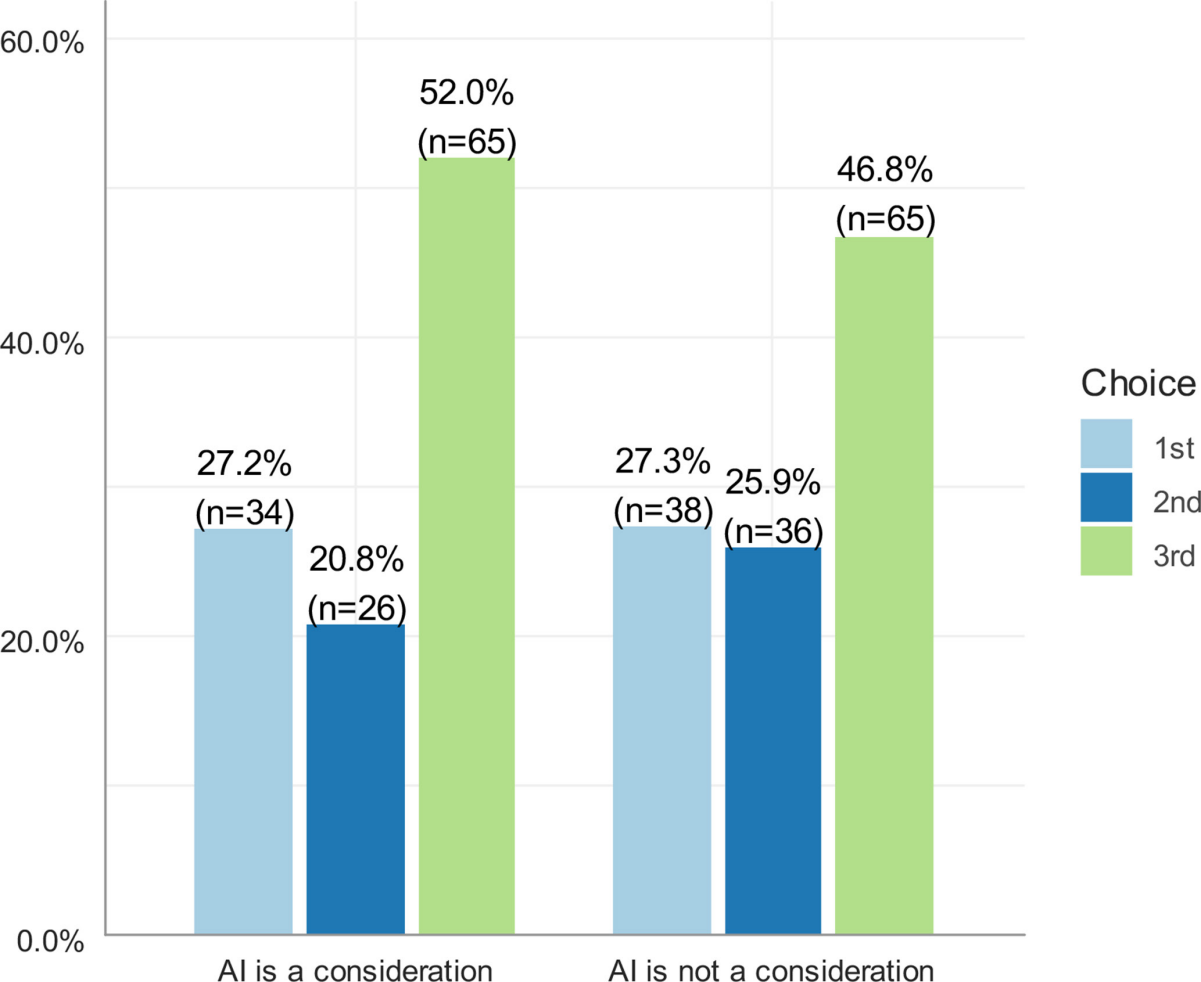
Respondents choice of radiology as a future career based on the impact of AI

When students were asked to rank their choice of radiology based on the impact of AI, the number of respondents who ranked radiology as one of their top three specialty choices increased from 125 to 139 (Figure 4). However, statistical analysis using the chi-square test of independence showed that the increase was not statistically significant (X^2^ = 0.886, *p* > 0.05).

The chi-square test of independence showed that choosing radiology as a future career was significantly associated with the potential impact of AI (X2 = 116.95, *p* < 0.001). Table 4 shows that, to some extent, consideration of the impact of AI discouraged students from ranking radiology as highly as they would have if AI was not a consideration. Of those who ranked radiology as their first choice when AI was not a consideration (*n* = 38), 13.2% initially ranked it as their third choice, 7.9% ranked it as their second choice, and 5.3% ranked it as lower than their third choice. Of those who ranked radiology as their second choice when AI was not a consideration (*n* = 36), 22.22% initially ranked it third, 8.33% ranked it lower than third, and 13.89% were initially not interested. Similarly, 26.15% of participants who ranked radiology as lower than third when AI was not a consideration initially ranked it as lower than third when AI was a consideration.

**Table 4.** Cross-tabulation of specialty choices based on the potential consideration of AI

		AI is not a consideration (*N* = 139)		
		first choice *N* = 38	second choice *N* = 36	third choice *N* = 65
AI is a consideration	first choice	28 (73.68%)	3 (8.33%)	1 (1.54%)
	second choice	3 (7.9%)	17 (47.22%)	3 (4.62%)
	third choice	5 (13.2%)	8 (22.22%)	39 (60%)
	Lower than third	2 (5.3%)	3 (8.33%)	17 (26.15%)
	Not interested	0 (0%)	5 (13.89%)	5 (7.69%)

**Figure 5.**
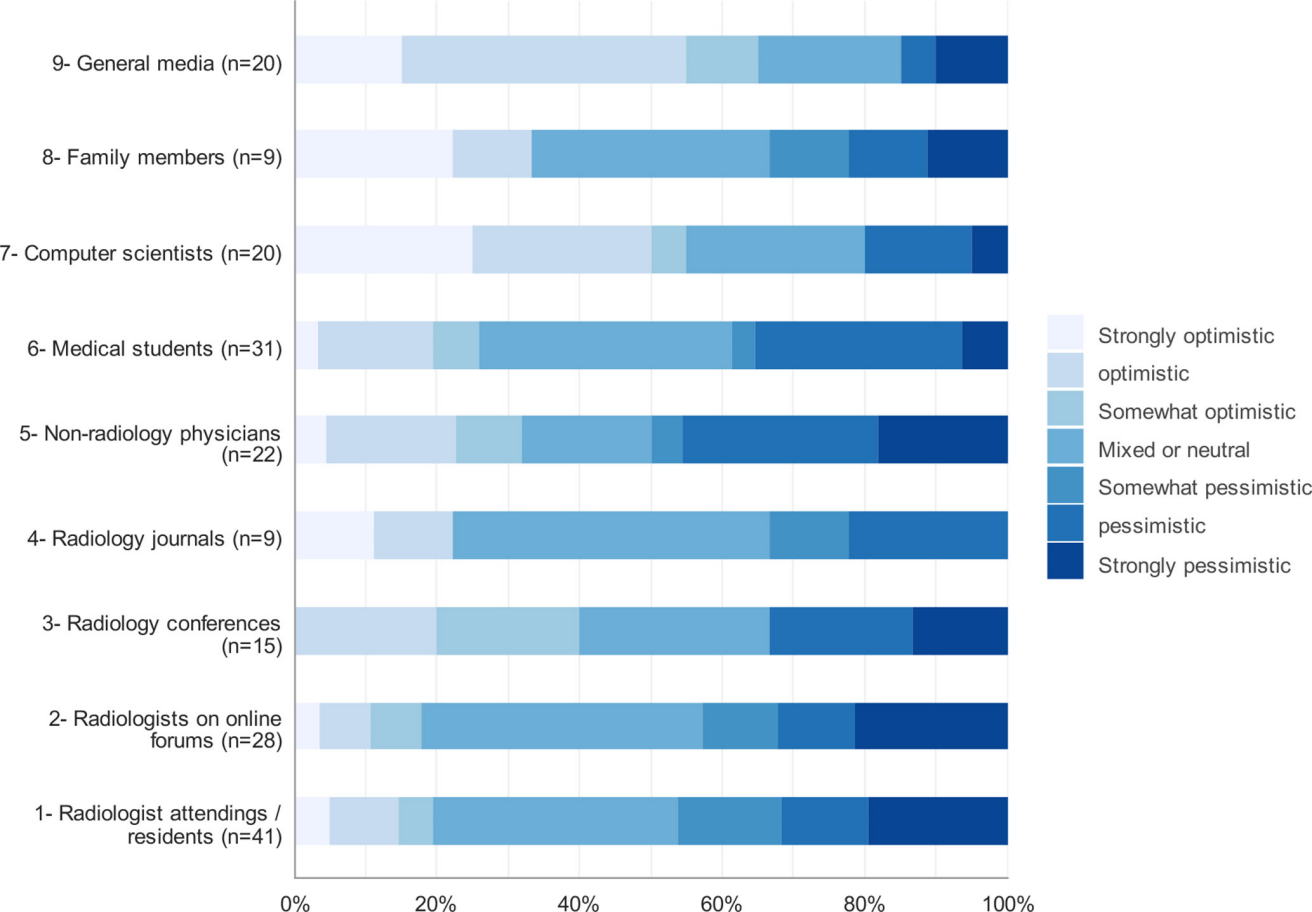
View of the impact of AI on radiology by source

Medical students were asked to list the sources from which they heard opinions about the impact of AI on radiology. They were also asked to state the influence (slider scale, 0 = no influence, 100 = extremely influential) of this source on their preference for radiology as well as the source’s overall view of the impact of AI on radiology. We analyzed the responses from the 139 students who would have ranked radiology as one of their top three specialty choices if AI was not a consideration. The most common sources (Figure 5) were radiologist attending/residents (*n* = 41), medical students (*n* = 31), and non-radiology physicians (*n* = 22). The least common sources of impact were family members (*n* = 9) and radiology journals (*n* = 9). The most influential source was radiology conferences (median = 70, *n* = 15). The median influence score was 60 for radiologists attendings/residents (*n* = 41), radiologists on online forums (*n* = 28) and radiology journals (*n* = 9).

**Figure 6.**
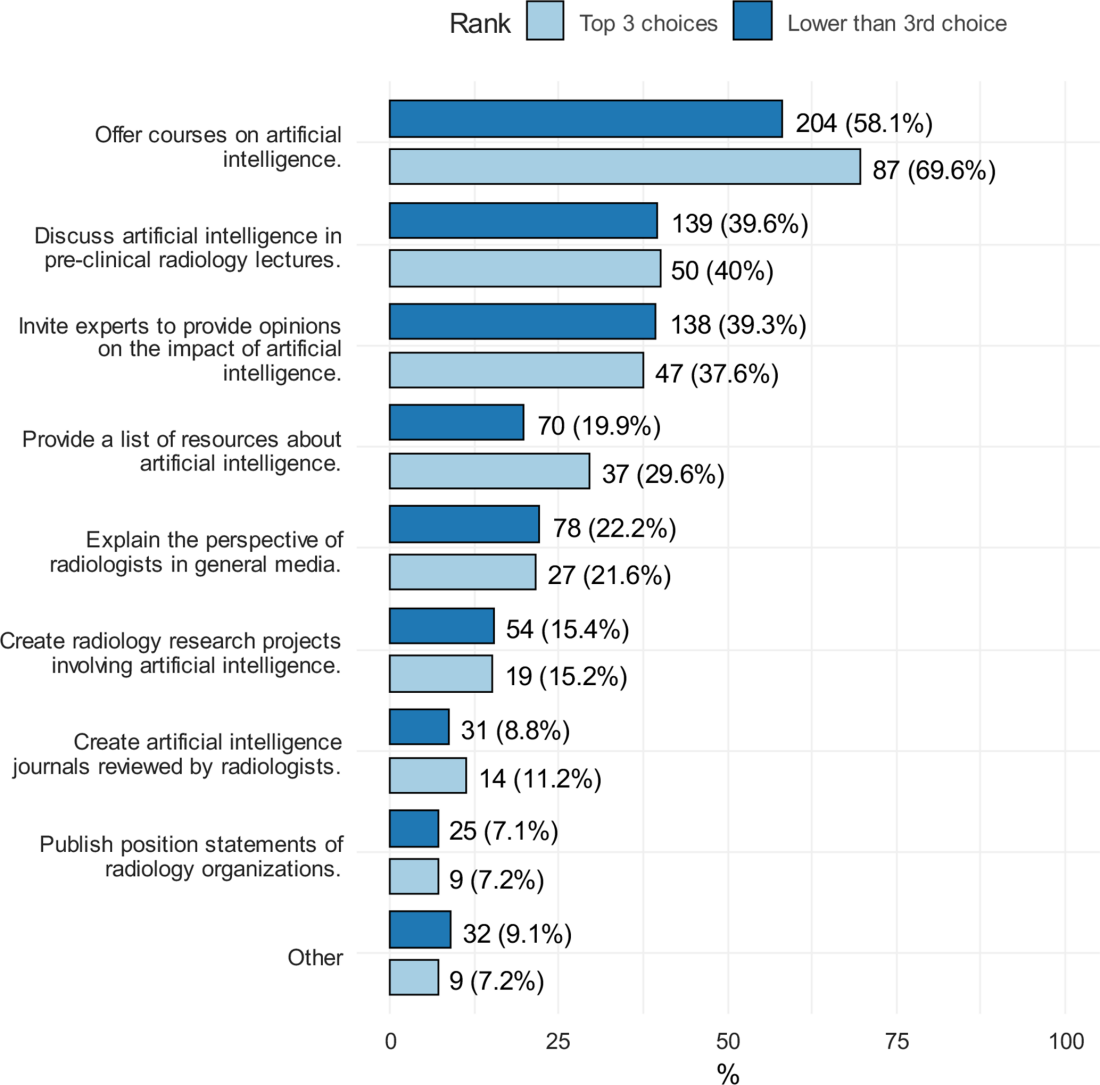
Respondents choices if the initiatives that can help medical students make informed decision regarding the impact of AI on radiology

The respondents were asked to select up to three suggestions that could aid the medical students make informed specialty decisions regarding AI and its possible impact when students are considering radiology as a career choice (Figure 6). Participants were classified into two groups based on their ranking of radiology: one of the top three choices (*n* = 125) and below third choice (*n* = 351).

## Discussion

This cross-sectional survey-based study was designed to determine the influence of AI on medical students choosing radiology as a career in Saudi Arabia and their perceptions of AI. It also aimed to determine the students’ background regarding AI and how much it can affect their interest in radiology as a career. Our study used a survey that was previously developed by Gong *et al*. in 2019.^14^

In the present study, among the respondents who ranked radiology as their first, second, or third career choice, 52% disagreed that AI will replace radiologists during their lifetime, and 44.8% agreed that AI will decrease the number of radiologists in the future. In Gong et al.’s study, 58.6% disagreed that AI will replace radiologists, and 67.7% agreed that AI will decrease the number of radiologists needed in the future.^14^ In addition, a German study on medical students conducted by Pintos Dos Santos et al revealed that 82.9% of respondents disagreed that AI will replace radiologists in the future.^19^

In the present study, 58.8% of respondents who ranked radiology as their first career choice were anxious about the uncertain influence of AI on radiology, as were 69.3% of respondents who ranked radiology as their second choice and 66.4% of respondents who ranked it as their third choice. In contrast, Gong et al found that 56.4% of their participants (all three groups merged) were anxious about the uncertain influence of AI on radiology. They also found that previous exposure to radiology was associated with lower agreement with the anxiety statement.^14^ In our study, respondents who believed they had a good understanding of AI were more likely to disagree with the anxiety statement. This indicates the importance of discussing the possible impact of AI on radiology to medical students, which can lead to greater understanding of this matter and assist the students in making a fair decision about their interest in radiology.

Interestingly, in our study, about 50% of our students agreed with a statement claiming that they have a good understanding of AI. However, when knowledge of AI was evaluated using five true/false questions, on average, about only one question was answered correctly per respondent, and 54.4% of the respondents did not answer any of the five questions correctly. These results contradict the self-reported knowledge results, confirming that there is a low level of knowledge regarding AI among medical students in the study sample.

Furthermore, this study showed a significant association between prior exposure to AI (through courses or research projects) and answering at least one question correctly. On the contrary, Gong et al showed that 78.9% of their respondents claimed to have a good understanding of AI, and when their knowledge of AI was tested using the five questions, the respondents achieved an average of 45.2% correct answers, however, 30.7% of their respondents did not answer any of the questions correctly.^14^ They also found that previous exposure to AI was significantly associated with higher knowledge towards AI.^14^ In Sit et al study, about half of their students obtained AI-related education, and students who did not obtain such education were significantly less likely to consider radiology as a career.^20^

A study by Collado-mesa et al that included 69 radiologists and radiology residents in the US showed that 36.2% of participants expressed doubts and reported that they may have changed their mind about continuing a career in radiology if they had known about the possible influence of AI.^22^ Previous research on radiology residents indicated an association between dissatisfaction with career choice and low sense of personal accomplishment -one of three burnout domains-.^23^ Therefore, it is important not to only educate students about the possible impact of AI but also give attention to radiology residents. The authors of this study encourage researchers to conduct future investigations into radiology residents’ perceptions of the possible impact of AI, how these perceptions can affect the residents, and the potential association with burnout, especially the personal accomplishment component.

In our study, 125 respondents chose radiology as one of their top three specialty choices. When asked to re-rank radiology as if AI was not a consideration, the number of interested respondents increased to 139. These results indicates that consideration of the potential impact of AI can discourage some students from selecting radiology as one of their top three specialty choices. Similarly, in Gong et al.’s study, the number of respondents interested in radiology increased from 133 to 160 when asked the same question.^14^ About half of students in Sit et al study in the U.K.^20^ and in Park et al study in the U.S.^24^ stated they will less probably consider radiology due to worries of AI.

The most common sources from which respondents heard from about the impact of AI on radiology were radiologists and radiology residents, followed by their colleagues’ medical students and non-radiology physicians. The respondents were asked to rate the influence of these sources on them. The results showed that radiology conferences had the most influence, non-radiology physicians and medical students had the least influence, and the rest of the sources were equally influential. The most common sources in Gong et al.’s study were radiologists and medical students, followed by non-radiology physicians and radiologists.^14^ Radiology conferences had the most influence on the students in Gong et al study, while medical students and the general media had the least influence.^14^ Majority of students in Pintos Dos Santos et al^19^ and Park et al^24^ studies received their knowledge of AI from social media.

In our study, students were asked to identify suggestions that could be made by radiology societies to aid medical students in making a decision about radiology as a career in light of the potential impact of AI. Offering a course on AI was the most common suggestion, followed by discussing AI in radiology lectures. In Gong et al.’s study, discussing AI in radiology lectures and inviting experts to discuss their opinions on the possible impact of AI in radiology were the most common suggestions.^14^

The main results of this study are generally in agreement with previously published studies in Canada,^14^ Germany,^19^ United Kingdom,^20^ Brazil^21^ and U.S.^24^ which concluded that AI has a negative influence on student choice for radiology. This survey was critical to conduct in our country and is still needed to conduct in other parts of the world, in order to understand if the anxiety towards AI exists and to provide a specific recommendation needed by each country or institute since the recommendations provided by earlier studies (including ours) may not be applicable to different populations. Although we did use a previous questionnaire developed by Gong et al^14^ the authors of this study assumed that Gong et al questionnaire was well designed and opted to use their questionnaire rather than developing a new questionnaire in order to compare our data with their findings and address the differences to provide our recommendations that fits our population.

However, the questionnaire could have been further modified to more fit our population, for example, how much AI is used in the country to the respondent’s knowledge and if it could influence the students and it is one of the limitations of this study. Moreover, there is a possibility that exposure to AI varies between respondents who answered that they had previous exposure, which can limit generalizability of the results. Furthermore, as a survey-based study, one of the main limitations is response bias.

The findings of this study can help radiology societies and academic radiologists understand the extent to which AI can discourage students from considering radiology and address the knowledge gap between students regarding AI and what could be done to fill that gap.

In conclusion, this study found that AI has a negative influence on medical students when making a decision about radiology as a career due to concerns that AI might displace radiologists in the future. Academic radiologists and medical schools are encouraged to educate their students about AI and its possible impact in order to aid students who are interested in radiology as a lifetime career. Investing in AI education is needed at an institute levels since radiology is not the only healthcare specialty impacted by AI.
